# Knowledge Translation in Healthcare – Towards Understanding its True Complexities: Comment on "Using Complexity and Network Concepts to Inform Healthcare Knowledge Translation"

**DOI:** 10.15171/ijhpm.2017.111

**Published:** 2017-09-12

**Authors:** Joachim P. Sturmberg

**Affiliations:** University of Newcastle, Callaghan, NSW, Australia.

**Keywords:** Complexity of Knowledge, Knowing in Medicine, Evidence, Complex Adaptive Organisation, Knowledge Transfer, Deviant Behaviour

## Abstract

This commentary argues that to fully appreciate the complexities of knowledge transfer one firstly has to distinguish between the notions of "data, information, knowledge and wisdom," and that the latter two are highly context sensitive. In particular one has to understand knowledge as being personal rather than objective, and hence there is no form of knowledge that a-priori is more authoritative than another. Secondly, knowledge transfer in organisations can only be successful if the organisation is organised and managed as a "complex adaptive organisation" – its key characteristics arising from it’s a-priori defined common "purpose, goals and values." Knowledge transfer, seen as "whole of system/organisation learning," is highly context sensitive; while the principles may apply to many organisations, knowledge as such is not transferable from one context to another, it always will be a unique learning exercise at this particular point in time in this particular organisation.

The paper by Kitson et al^1^ is commendable as it challenges the ways we think about knowledge translation in the healthcare context. Complexity and network understandings clearly offer a more useful framework to appreciate the multiple dynamics impacting on the successful implementation of new knowledge into clinical care.

However, the two most important aspects to understand the difficulties of translating new knowledge in established healthcare organisations – the complex nature of knowledge, and the key behaviours of complex adaptive organisations – have largely been brushed over.

This commentary explores the complex adaptive nature of knowledge and its relationship to “evidence”; and it alludes to the importance of appreciating “purpose, goals, and values” as the foundational elements for an organisation to become a seamlessly integrated organisational system.

## Knowledge Is More and Different to the Sum of its Constituent Parts

Knowledge and information are frequently used interchangeably in discourses about knowledge management and knowledge translation into clinical practice. This is most evident in statements like those quoted by Kitson et al^1^ – “*A 1998 landmark study reviewing the quality of care in the United States indicated that some 30% to 50% of care delivery was not in line with best available evidence*.”^2^

## Data – Information – Knowledge – Wisdom

The struggle to distinguish between data, information, knowledge and wisdom is a long-standing one (see Box 1).

**Box 1. Perspectives on “Knowledge”**

√ Data is not information, information is not knowledge, knowledge is not understanding, understanding is not wisdom. ***Clifford Stoll***
√ Contrary to the old cliché, facts do not speak for themselves. Facts are chameleons whose shape and color reflect their handlers. A fact is only a piece of information. ***The Blog @ Evidence Explained***
√ Information is not knowledge. ***Albert Einstein***
√ Where is the life we have lost in living? Where is the wisdom we have lost in knowledge? Where is the knowledge we have lost in information? ***TS Eliot***

### Data and information

*Data* are simple facts, like a biochemistry result or a population’s morbidity; linked data create *information* like the relationship between a biochemical parameter and a population’s morbidity. Both are reductionist in nature, and both are perceived as facts providing a sense of “certainty.”

### Knowledge

*Knowledge*, on the other hand, is *emergent* and *contextual* in nature^3^ (also referred to as situated^4^). Knowledge arises on seeing multiple data and pieces of information within a contextual network and from within one’s own frames and experiences. These insight led Michael Polanyi^5^ to the conclusion that *knowledge is personal* (colloquially stated as “I know”).

*Knowledge* can be divided into *knowing what* – naming facts and relationships – and *knowing how* – explaining procedures. In addition, knowledge can be separated into *explicit* knowledge which can be codified and hence easily communicated, and *tacit* knowledge which cannot be codified and can only be transferred through shared experiences.^6^

**Figure 1:**
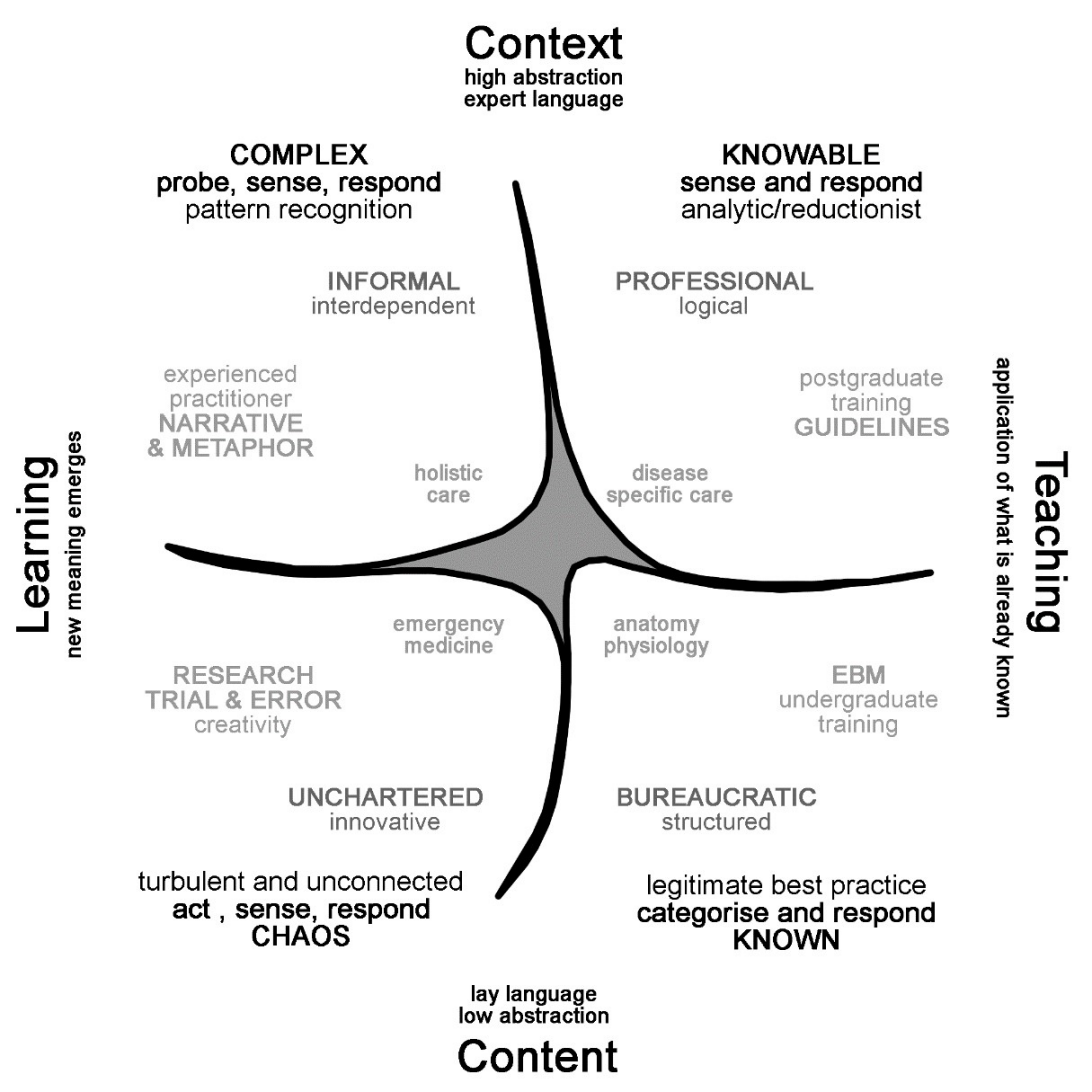
The Cynefin Framework of “Knowing in Medicine.”
The Cynefin model visualises the 4 domains of knowledge and their main characteristics. Of note, no single domain has any more authority over any other, ie, knowing and knowledge are value-neutral. However, appreciating each knowledge domain facilitates an understanding of the whole. Equally, note the differences in knowledge transfer, things on the right can be thought, things on the left have to learned through experiences in context (first published in Sturmberg and Martin^6^).

Moreover, knowledge generation is an iterative process amongst all members of an organisation. As Snowden emphasised, this requires *conversations* that facilitate *sense-making* – what does the available data and information *mean* in *our specific context*.^7^ The *contextual* nature of medical knowledge within the Cynefin framework is depicted in Figure 1 – note how each of the 4 domains of “knowing” influences medical worldviews and practices, and how each domain is associated with a different level of *certainty* (the narrow focus of the 2 right-hand domains entails a high level of certainty – they are more stable and amenable to “semi-reductive approaches” if context is fully taken into account; whereas the 2 broad domains on the left-hand side entail high levels of uncertainty– they are more unstable and highly context sensitive, their behaviours are not predictable and outcomes can only be observed in an anticipatory/emergent fashion).^6^

### Wisdom

**Figure 2:**
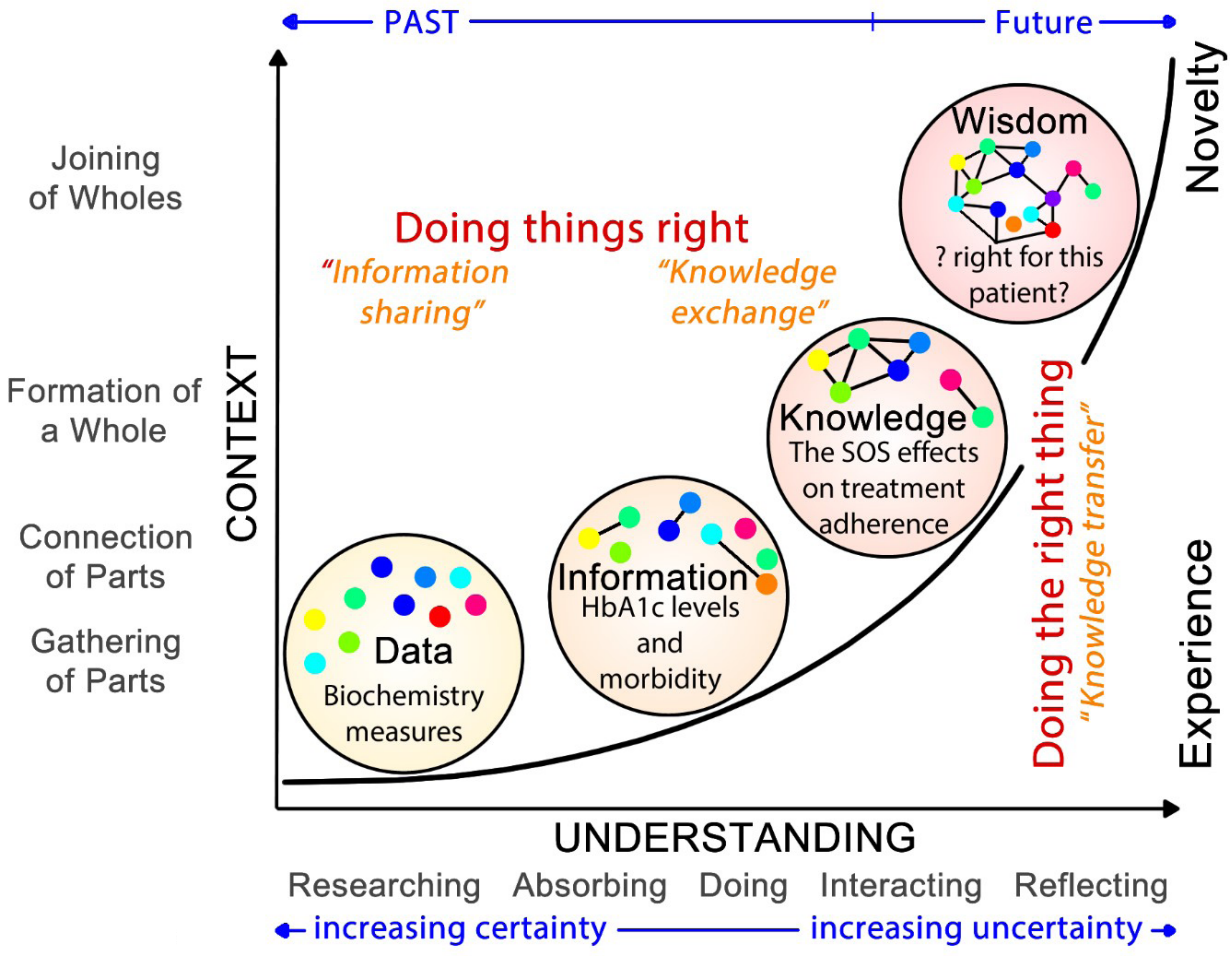
Distinguishing Data, Information, Knowledge and Wisdom Through Different Lenses.
Note the increasing levels of understanding on the x-axis, associated with an increase in the levels of uncertainty from data to wisdom. Also note the increasing contextualisation from data to wisdom along the y-axis. The two dimensions have a nonlinear relationship.

*Wisdom* is the ability to synthesise “all sorts of knowledge” and “prior experiences” in “the context of a particular situation.” Being able to see the *whole picture* and being able to see the *best possible decision under the circumstances* distinguishes knowledge transfer as a “mechanistic” process from that of “consciously sharing” insights (Figure 2).

Superimposed are the temporal aspects between data and information as things arising in the past, whereas knowledge, to a lesser extent, and wisdom are emergent, resulting in novel insights.

In Peter Drucker’s terms, data, information and knowledge are required *to do things right*, but it is wisdom that leads to *doing the right thing*.

## The Ambiguities of Evidence and Knowledge

Evidence needs to be distinguished from belief – or as David Hume put it - *A wise man proportions his belief to the evidence. Evidence* in its most basic form is defined as *that which justifies belief.*^8^ The *scientific method* is generally regarded as the way to generate the evidence that *verifies* or *refutes* a hypothesis based on:

- observations of phenomena that occur in the natural world, or
- observations that are created through experiments.^9^

These scientific approaches aim to avoid bias, the “prejudicial attribution” of observations according to one’s preconceived ideas.

## The Problems With Evidence

While theoretically sound, the scientific method has fundamental problems. Firstly, as Popper emphasised: by choosing what to observe, we also decide what not to observe,^10^ and secondly, any single contradictory observation refutes a hypothesis.^10^ Popper argued from within the dominant reductionist paradigm of his time, and he probably could not have foreseen how much more relevant his arguments would be for a nonlinear complex adaptive understanding of the world.

In health research, we constantly narrowly define what to observe (and by implication what not), like “cardiac death increases with cholesterol levels.” This type of research evokes a sense of certainty about the cause of cardiac death, where in fact it only alludes to a correlation between two data. In addition, much of what we really want to observe is not directly measurable, we therefore replace those with surrogate measures^11-13^; as Krumholz and Lee highlighted: *we accept a change in a biomarker as a perfect proxy for patient benefit*^14^ (low cholesterol levels equals low cardiac mortality). And again, this type of simplification aims to provide reassurance to both, patients and doctors, and by way of the “evidence-based doctrine” falsely asserts professional and regulatory authority.^15^

However, these “reductionist” approaches fail Popper’s basic dictum that any single contradictory observation falsifies a hypothesis. That there are plenty of contradictory observations in health should not be surprising as natural phenomena “as a rule” have a long-tail (or nonlinear, Pareto) distribution pattern. Contradictory and thus refuting observations typically “hide” in the long tail of the distribution curve – contrary to the “traditional reductionist viewpoint, they are not outliers but “part of the normal spectrum.”^16,17^

## Likelihood and Confidence Intervals – Proxies of Evidence in a Complex Adaptive World?

Much of the research concerned with natural world phenomena – biology, health and disease, psychology or social sciences – looks at *associations* between phenomena as potential “pointers to” *causal pathways*. However, associations **never** establish *proof of evidence*.

It therefore is of utmost importance to understand that the prevailing concept of “evidence being “established” if observations show a likelihood of *not having occurred by chance* based on *probability statistics* and the *95% confidence interval* is flawed, and at best can be described as a “downgraded” concept of evidence.” Likelihood-ratios of association are simply that – likelihoods or probabilities – they do not have the *authority* to demand generalisation for action in a complex adaptive world.

## Can Knowledge Count as Evidence?

As *knowledge is a personal construct* (“I know”),^5^ one would – *a-priori* – have to conclude that knowledge cannot count as evidence. However, as our knowledge arises from our personal learning in our unique context, the statement “*I know” nevertheless* fulfils the evidence criterion of “*that which justifies belief*.”^8^

### Evidence and Knowledge – a Circular Argument

We are left with an infinite conundrum – is “*that which justifies belief*” to be regarded as “objective” evidence or merely as “subjective” knowledge. Evidence creation is based on “subjective” assumptions – namely our *a-priori* “subjective” knowledge within the context of our worldview, and knowledge is shaped by the “seemingly objective nature” of evidence as defined within this worldview.

**Figure 3:**
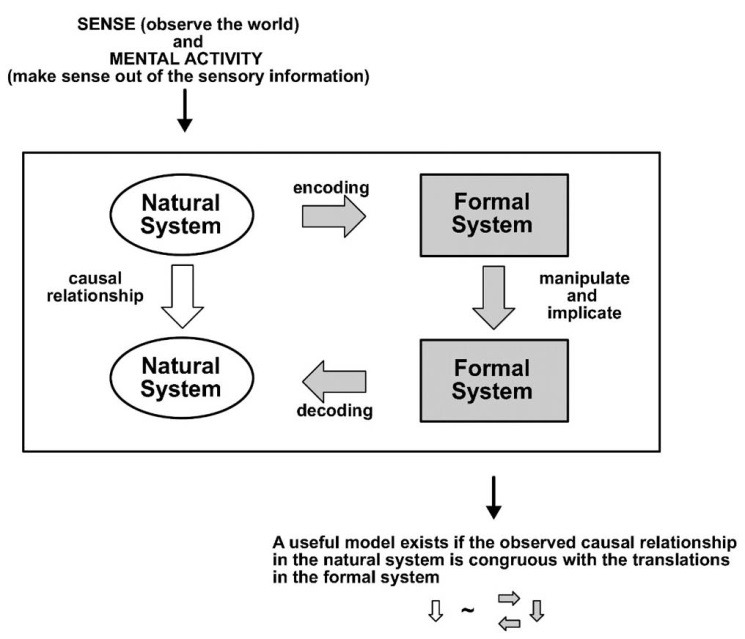
The Scientific Process of Taking Observations From in the Real World Into the Scientific World and Back Into the Real World. There is ample scope for observer bias and the observer’s mental worldview to “manipulate” the real world based on “scientific” argument (first published in Sturmberg^19^).

Rosen^18^ first explored the relationships between the “observable reality” and its representation in “scientific models.” He suggested that the scientific process, despite its aim to prevent observer bias, entails a person translating the “natural (real world) system” into a “formal (scientific) system” that can be evaluated and manipulated; conclusions reached in the “formal (scientific) system” are subsequently translated back into the “natural (real world) system” (Figure 3). Expanding on these insights Box^20^ coined the phrase “Essentially, all models are wrong, but some are useful.”

## Knowledge Translation

Kitson et al^1^ rightly point to the difficulties of knowledge translation across organisational boundaries – this requires an “organisation-wide” approach. Organisations at large are linear hierarchies, an organisational structure that stands in the way of allowing effective network relationships to emerge. Effective knowledge translation across organisational boundaries requires a shift from *linear* hierarchical to *dynamic complex adaptive* networked organisations.

## What Is a Complex Adaptive Organisation?

Organisations are defined as “a group of people working together with a particular purpose.” For an organisation to be a *dynamic complex adaptive organisation* it needs to define, *a-priori,* not only its purpose, but also its *specific goals and values*. If those are shared and understood by all of its members they become the organisation’s “driver,” a prerequisite to function seamlessly across and between its various “organisational levels.” It is the organisation’s driver that “determines” (the term is used in a literal sense) the configuration of its agents and their interactions (behaviours). Interactions facilitate *learning* – learning is the key feature that distinguishes a complex adaptive system from a “simple” complex system.^21^

## Knowledge translation: Learning in complex adaptive organisations

Knowledge translation mandates “learning of the whole organisation.” As a *whole of organisation* effort, it requires an environment that not only accepts the *context sensitivity* of knowledge and evidence but also denies one form of “knowledge and evidence” an *a-priori* greater authority over any other. It must be emphasised that even the “best knowledge and evidence” remains open to scrutiny, it is not the “truth.”^15^

*What we know* and *how we know* emerges over time in the context of our work.^3^ *Emergence* is a key phenomenon of complexity that is highly sensitive to its *starting conditions* – the reason why solutions invariably cannot be successfully transferred from one organisational setting to another. Emergent processes result in recognisable pattern formations – eg, not every patient with angina responds to the same treatment in exactly the same way; and the outcomes of care for ischaemic heart disease between socioeconomically diverse cohorts varies widely. The pleural – pattern formations – is key; patterns reflect outcomes that are *similar but not the same*, and at the same time, these outcomes are *mutually agreeable*, in other words, each outcome reflects the *most adapted responds* under the given local conditions.

Knowledge thus entails a level of *uncertainty* that is not present in its constituent parts, ie, data and information.

Truly complex adaptive organisations indeed understand the temporal nature of knowledge and evidence, and constantly seek new observations and reflections – both utilising linear and nonlinear approaches in their appropriate context – to *create* new “knowledge and evidence” in light of newly arising problems.

## Deviant Behaviour Is Neither Irrational nor Ignorant

By implication, the statement that “*A 1998 landmark study reviewing the quality of care in the United States indicated that some 30% to 50% of care delivery was not in line with best available evidence*”^2^ means that these 30%-50% of healthcare providers are either irrational or ignorant. This is a classical decontextualized and reductionist viewpoint, based in only seeing data and information without appreciating context. Context determines which part of the “known” knowledge base is applicable,^3^ and what appears to be deviant behaviour in most cases is nothing less than the judicious application of this knowledge base in this particular context.^20^

This point has been succinctly highlighted by Peter Drucker who pointed to the distinction between *doing things right* (as in adhering to EBM-guidelines) and *doing the right thing* (as in adapting interventions and treatments in light of *this person’s* needs and context).

As Kitson et al^1^ rightly state: “*The biggest challenge is to move away from the security of the linear-rational thinking into acknowledging that life is much more complex and unpredictable. It is only when people sit together and engage in these conversations that the true synergies emerge. Paradoxically, creativity and curiosity are the true innovators in science.*”

## Ethical issues

Not applicable.

## Competing interests

Author declares that he has no competing interests.

## Author’s contribution

JPS is the single author of the paper.
